# Assessment of Suicide in Japan During the COVID-19 Pandemic vs Previous Years

**DOI:** 10.1001/jamanetworkopen.2020.37378

**Published:** 2021-02-02

**Authors:** Haruka Sakamoto, Masahiro Ishikane, Cyrus Ghaznavi, Peter Ueda

**Affiliations:** 1 Department of Health Policy and Management, Keio University, Tokyo, Japan; 2 Department of Global Health Policy, University of Tokyo Graduate School of Medicine, Tokyo, Japan; 3 Disease Control and Prevention Center, National Center for Global Health and Medicine, Tokyo, Japan; 4 Department of Medicine, Washington University School of Medicine in St Louis, St Louis, Missouri; 5 Clinical Epidemiology Division, Department of Medicine, Solna, Karolinska Institutet, Stockholm, Sweden

## Abstract

**Importance:**

There are concerns that suicide rates may have increased during the coronavirus disease 2019 (COVID-19) pandemic.

**Objective:**

To assess whether suicide rates in Japan increased in April through November 2020 compared with previous years.

**Design, Setting, and Participants:**

This cross-sectional study used national data obtained from the Ministry of Health, Labor and Welfare from 2016 to 2020 on the monthly number of individuals who died of suicide in Japan from January to November of 2016 to 2020.

**Exposure:**

2020 vs previous years.

**Main Outcomes and Measures:**

The main outcome was monthly suicide rates, calculated as the number of individuals who died of suicide divided by the total population. A difference-in-difference regression model was used to estimate the change in monthly suicide rates in April to November 2020 vs these months in 2016 to 2019.

**Results:**

Analyses included 90 048 individuals (61 366 [68.1%] men) who died of suicide from 2016 to 2020. The difference-in-difference analysis of men showed that there was no increase in suicide rates from April through September 2020 compared with these months in 2016 to 2019, but that suicide rates were increased in October (difference-in-difference, 0.40 [95% CI, 0.14 to 0.67] suicide deaths per 100 000 population) and November (difference-in-difference, 0.34 [95% CI, 0.07 to 0.60] suicide deaths per 100 000 population). Among women, suicide rates in 2020 compared with 2016 to 2019 increased in July (difference-in-difference, 0.24 [95% CI, 0.09 to 0.38] suicide deaths per 100 000 population), August (difference-in-difference, 0.30 [95% CI, 0.16 to 0.45] suicide deaths per 100 000 population), September (difference-in-difference, 0.29 [95% CI, 0.15 to 0.44] suicide deaths per 100 000 population), October (difference-in-difference, 0.62 [95% CI, 0.48 to 0.77] suicide deaths per 100 000 population), and November (difference-in-difference, 0.29 [95% CI, 0.15 to 0.44] suicide deaths per 100 000 population). In secondary analyses in which the suicide rates of 2020 were compared with the expected rates based on trends from 2011 to 2019, the increases in suicide rates were most pronounced among men aged younger than 30 years (eg, November: observed vs expected rate ratio [RR], 1.48 [95% CI, 1.26-1.71]) and women aged younger than 30 years (eg, October: observed vs expected RR, 2.14 [95% CI, 1.76 to 2.52]) and 30 to 49 years (eg, October: observed vs expected RR, 2.30 [95% CI, 2.01 to 2.58]).

**Conclusions and Relevance:**

These findings suggest that compared with previous years, suicide rates in Japan in 2020 increased in October and November for men and in July through November for women.

## Key Points

**Question:**

Have suicide rates in Japan increased during the coronavirus disease 2019 (COVID-19) pandemic?

**Findings:**

In this cross-sectional study using national data on suicide mortality in Japan, including 90 048 individuals who died of suicide, suicide rates in 2020 compared with 2016 to 2019 were increased in October and November for men and in July through November for women, and the relative increases were particularly pronounced among men aged younger than 30 years and women aged younger than 30 years and 30 to 49 years.

**Meaning:**

These findings suggest that the COVID-19 pandemic was associated with increases in suicide rates in Japan.

## Teaser

This cross-sectional study examines suicide rates in Japan during the coronavirus disease 2019 (COVID-19) pandemic in 2020 compared with 2016 to 2019.

## Introduction

Measures taken to mitigate the coronavirus disease 2019 (COVID-19) pandemic have raised concerns that suicide rates might increase as a result of heightened economic stress, decreased human contact, and exacerbations of preexisting mental health conditions.^1,2,3^ While data from high-income countries indicate that there was no increase in suicide rates during the early months of the pandemic,^4^ a recent analysis from Japan^5^ using data on suicide rates from 2010 through September 2020 found an excess of suicide deaths among women, but not men, in July, August, and September 2020; data by age group were not presented. In this study, we assessed potential changes in suicide rates associated with the pandemic in Japan through November 2020 and performed analyses by sex, age group, and occupational status.

## Methods

Per policy at the University of Tokyo Graduate School of Medicine, ethical review and informed consent were not required because no individual level data were used and no data could be linked to any individual.^6^ This study is reported following the Strengthening the Reporting of Observational Studies in Epidemiology (STROBE) reporting guideline. We used national data from 2011 to 2020 on the monthly number of individuals who died of suicide from January through November (the latest available data for 2020) from the Ministry of Health, Labor, and Welfare, Japan.^7^ According to Japanese legislation, an autopsy performed by a physician is mandated for any death which is not known to have been caused by an organic disease. Deaths that are deemed to be suicide are reported within 1 day to the police authorities. National data on suicide are compiled by the National Police Agency and disseminated to the Ministry of Health, Labor and Welfare, which provides statistics on cause of death for all deaths in Japan.^8^

We assessed the association between the COVID-19 pandemic and suicide rates using 2 different methods. First we used a difference-in-difference analysis using data from 2016 through 2020 as our primary analysis. For secondary analysis, we performed a comparison of observed vs expected suicide rates accounting for trends in suicide rates since 2011.

Events potentially associated with adverse mental health outcomes, including an economic downturn and loss of employment,^1,2^ may have been introduced in association with stay-at-home orders issued by central and local governments in late March 2020 (the orders were lifted in May, although the public has since been encouraged to limit their social interactions during the pandemic) and the declaration of national emergency on April 7 (lifted on May 25).

### Statistical Analysis

In the difference-in-difference analysis, we assessed differences in suicide rates in April through November 2020 compared with 2016 through 2019. For each sex, age group (all ages, <30 years, 30-49 years, 50-69 years, or ≥70 years) and month, we calculated the suicide rate by dividing the number of individuals who died of suicide by the total population in each age group using data from the Population Census of Japan. We estimated the difference in suicide rates in 2020 vs 2016 through 2019 using a regression model with the suicide rate as the dependent variable; independent variables included a variable for each month, a variable representing the mean difference in suicide rate per month for 2020 vs 2016 through 2019 in January through March (a crude measure of factors associated with suicide rates that may be specific to 2020 but unrelated to the pandemic), and interaction variables for each month of April through November and 2020 (difference-in-difference). The difference-in-difference was considered as statistically significant if the 95% CI did not overlap 0. We also performed difference-in-difference analyses by occupational status of the individuals who died by suicide (classified as family business or self-employed, employed, student, homemaker [women only], or unemployed). Because changes in occupational status may be considered a downstream event of the pandemic and the measures introduced to contain it, we used the absolute number of individuals who died by suicide stratified by occupational status (rather than rates) as the outcome variable. For example, unemployment rates have increased in Japan during the COVID-19 pandemic^9^; as the number of individuals who are unemployed has increased, the results of the difference-in-difference model should not be considered as changes in suicide rates associated with the pandemic among individuals who are unemployed. Rather, by providing data on changes in the absolute number of individuals who committed suicide by occupational status category, this analysis aimed to explore possible mechanisms associated with potential changes in the suicide rates in 2020 vs 2016 to 2019.

In the secondary analyses, we accounted for the declining trend in suicide rates during the past decade in Japan.^10^ We used suicide rates from 2011 to 2019 to estimate the expected rate for 2020 and compared the observed vs expected suicide rates. To account for seasonal fluctuations in the number of suicides,^10^ we assessed trends in suicide rates for each month separately: for each month and sex, we estimated the yearly change in suicide rate between 2011 and 2019 using linear regression models with the logarithm of the suicide rate as the dependent variable and year as a continuous independent variable. We used these models to estimate the expected suicide rate in 2020 and calculated the ratio of observed vs expected suicide rates. We performed the analyses in the total age range and by age group. The difference between the observed and expected rate was considered as statistically significant if the 95% CI of the rate ratio (RR) did not overlap 1. Stata statistical software version 16.1 (StataCorp) was used for all analyses.

## Results

### Difference-in-Difference Analysis

**Figure 1.**
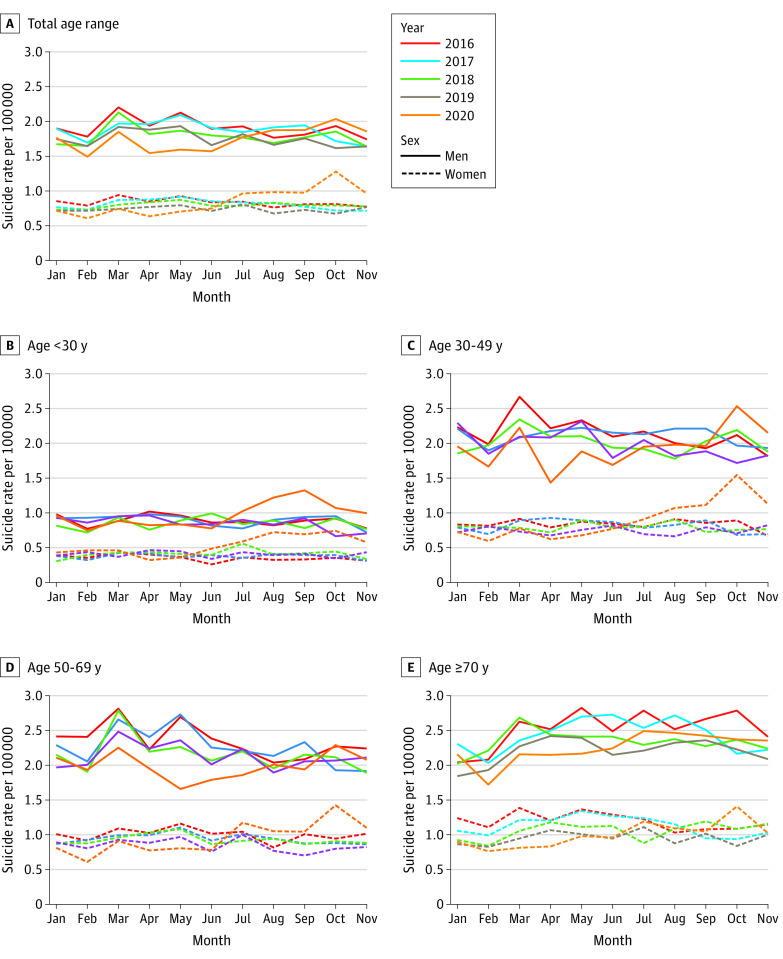
Monthly Suicide Rates in January to November in Japan in 2016-2020 by Age

In January through November, from 2011 through 2020, 90 048 individuals died of suicide in Japan, including 61 366 (68.1%) men and 28 682 (31.9%) women. Data on age were available for 61 135 men (99.6%) and 28 635 women (98.3%). Among men who died of suicide, 8536 (14.0%) were younger than 30 years, 18 979 (31.0%) were aged 30 to 49 years, 19 574 (32.0%) were aged 50-69 years, and 14 046 (23.0%) were 70 years or older. Among women who died of suicide, 3755 (13.1%) were younger than 30 years, 7395 (25.8%) were aged 30 to 49 years, 8585 (30.0%) were aged 50 to 69 years, and 8900 (31.1%) were 70 years or older. Monthly suicide rates for each year in the total age range and by age group are shown in Figure 1. Information about occupational status was available for 58 094 men (94.7%) and 27 122 women (94.6%). Among men who died of suicide, 5103 (8.8%) were self-employed or had a family business, 20 670 (35.6%) were employees, 4533 (7.8%) were students, and 2777 (47.8%) were unemployed. Among women who died of suicide, 697 (2.6%) were self-employed or had a family business, 4806 (17.7%) were employees, 2120 (7.8%) were students, 4724 (17.4%) were homemakers, and 14 775 (54.5%) were unemployed. Suicide rates were higher among men and those aged 30 years or older, and the numbers of individuals who died of suicide were higher among unemployed men, unemployed women, and employed men. (Figure 1; eFigure 1 in the Supplement).

The results of the difference-in-difference analysis for men are shown in Table 1. In the all ages group, there were no statistically significant differences in the suicide rate in April through September 2020 vs 2016 to 2019; however, the difference-in-difference was significantly increased in 2020 compared with 2016 to 2019 in October (2.03 vs 1.78 deaths by suicide per 100 000 population; difference-in-difference, 0.40 [95% CI, 0.14 to 0.67] deaths by suicide per 100 000 population), and November (1.85 vs 1.66 deaths by suicide per 100 000 population; difference-in-difference, 0.34 [95% CI, 0.07 to 0.60] deaths by suicide per 100 000 population). In analyses by age group, suicide rates in 2020 compared with 2016 to 2019 were decreased in April among men aged 30 to 49 years (1.43 vs 2.15 deaths by suicide per 100 000 population; difference-in-difference, −0.53 [95% CI, −0.94 to −0.12] deaths by suicide per 100 000 population) and in May among those aged 50-69 years (1.66 vs 2.52 deaths by suicide per 100 000 population; difference-in-difference, −0.62 [95% CI, −1.04 to −0.21] deaths by suicide per 100 000 population). Suicide rates were increased in July through November for men aged younger than 30 years, with difference-in-difference estimates ranging from 0.23 (95% CI, 0.03 to 0.43) deaths by suicide per 100 000 population in October to 0.47 (95% CI, 0.26 to 0.67) deaths by suicide per 100 000 population in September. Similarly, suicide rates among men aged 30 to 49 were increased in October (difference-in-difference, 0.71 [95% CI, 0.30 to 1.12] deaths by suicide per 100 000 population) and November (difference-in-difference, 0.46 [95% CI, 0.05 to 0.88] deaths by suicide per 100 000 population), and rates among those aged 50 to 69 were increased only in October (difference-in-difference, 0.43 [95% CI, 0.01 to 0.84] deaths by suicide per 100 000 population). No significant increase was observed for those aged 70 years or older.

**Table 1.** Individuals Who Died of Suicide, Suicide Rates, and Difference-in-Difference Analysis of the Suicide Rates in April Through November 2020 vs 2016-2019 in Men

Age group	Deaths, No. (rate per 100 000 population)^a^										
	Jan	Feb	Mar	Apr	May	Jun	Jul	Aug	Sep	Oct	Nov
**Total**											
2016-2019	1111 (1.80)	1042 (1.69)	1266 (2.06)	1170 (1.90)	1234 (2.00)	1117 (1.81)	1133 (1.84)	1083 (1.76)	1121 (1.82)	1096 (1.78)	1025 (1.66)
2020	1082 (1.77)	914 (1.49)	1133 (1.85)	946 (1.54)	976 (1.59)	962 (1.57)	1087 (1.77)	1148 (1.87)	1149 (1.88)	1246 (2.03)	1136 (1.85)
Difference-in-difference, deaths per 100 000 population (95% CI)^b^	NA	NA	NA	−0.21 (−0.47 to 0.06)	−0.26 (−0.53 to 0)	−0.1 (−0.36 to 0.17)	0.08 (−0.18 to 0.35)	0.26 (0 to 0.53)	0.2 (−0.06 to 0.47)	0.4 (0.14 to 0.67)	0.34 (0.07 to 0.60)
**<30 y**											
2016-2019	160 (0.91)	143 (0.82)	163 (0.93)	163 (0.93)	159 (0.91)	153 (0.88)	148 (0.85)	151 (0.86)	155 (0.89)	151 (0.87)	130 (0.74)
2020	166 (0.96)	129 (0.75)	153 (0.89)	142 (0.82)	144 (0.84)	134 (0.78)	177 (1.03)	211 (1.22)	229 (1.33)	185 (1.07)	172 (1.00)
Difference-in-difference, deaths per 100 000 population (95% CI)^b^	NA	NA	NA	−0.09 (−0.29 to 0.12)	−0.05 (−0.25 to 0.15)	−0.08 (−0.28 to 0.12)	0.2 (0 to 0.41)	0.38 (0.18 to 0.59)	0.47 (0.26 to 0.67)	0.23 (0.03 to 0.43)	0.28 (0.08 to 0.48)
**30-49 y**											
2016-2019	366 (2.15)	329 (1.93)	392 (2.30)	366 (2.15)	383 (2.24)	340 (2.00)	353 (2.07)	333 (1.96)	344 (2.02)	341 (2.00)	318 (1.86)
2020	322 (1.96)	274 (1.67)	366 (2.22)	236 (1.43)	310 (1.88)	278 (1.69)	321 (1.95)	326 (1.98)	323 (1.96)	417 (2.53)	354 (2.15)
Difference-in-difference, deaths per 100 000 population (95% CI)^b^	NA	NA	NA	−0.53 (−0.94 to −0.12)	−0.18 (−0.59 to 0.23)	−0.13 (−0.54 to 0.28)	0.06 (−0.35 to 0.47)	0.2 (−0.21 to 0.61)	0.12 (−0.29 to 0.53)	0.71 (0.30 to 1.12)	0.46 (0.05 to 0.88)
**50-69 y**											
2016-2019	363 (2.21)	344 (2.10)	442 (2.69)	373 (2.27)	413 (2.52)	358 (2.18)	365 (2.22)	330 (2.01)	354 (2.16)	344 (2.10)	335 (2.04)
2020	337 (2.11)	309 (1.94)	360 (2.25)	312 (1.95)	265 (1.66)	286 (1.79)	297 (1.86)	321 (2.01)	310 (1.94)	366 (2.29)	332 (2.08)
Difference-in-difference, deaths per 100 000 population (95% CI)^b^	NA	NA	NA	−0.08 (−0.50 to 0.33)	−0.62 (−1.04 to −0.21)	−0.16 (−0.57 to 0.26)	−0.13 (−0.55 to 0.29)	0.23 (−0.18 to 0.65)	0.01 (−0.40 to 0.43)	0.43 (0.01 to 0.84)	0.27 (−0.15 to 0.69)
**≥70 y**											
2016-2019	220 (2.05)	221 (2.07)	266 (2.49)	265 (2.47)	276 (2.58)	261 (2.44)	262 (2.45)	266 (2.48)	262 (2.45)	255 (2.38)	240 (2.24)
2020	251 (2.16)	200 (1.72)	251 (2.16)	250 (2.15)	252 (2.17)	261 (2.25)	290 (2.50)	287 (2.47)	282 (2.43)	276 (2.38)	274 (2.36)
Difference-in-difference, deaths per 100 000 population (95% CI)^b^	NA	NA	NA	−0.13 (−0.65 to 0.39)	−0.23 (−0.75 to 0.29)	−0.01 (−0.53 to 0.51)	0.23 (−0.29 to 0.74)	0.17 (−0.35 to 0.69)	0.16 (−0.36 to 0.68)	0.17 (−0.34 to 0.69)	0.31 (−0.21 to 0.82)

Abbreviation: NA, not applicable.

^a^ Excluding 602 men who died of suicide for whom information about age was not available.

^b^ Subtraction of differences in January through April from differences in April through September. Negative values represent lower suicide rates in 2020 vs 2016-2019.

The results of the difference-in-difference analysis for women are shown in Table 2. In the analysis of the all ages group for women, suicide rates in 2020 compared with 2016 to 2019 increased in July (0.96 vs 0.82 deaths by suicide per 100 000 population; difference-in-difference, 0.24 [95% CI, 0.09 to 0.38] deaths by suicide per 100 000 population), August (0.98 vs 0.77 deaths by suicide per 100 000 population; difference-in-difference, 0.30 [95% CI, 0.16 to 0.45] deaths by suicide per 100 000 population), September (0.97 vs 0.78 deaths by suicide per 100 000 population; difference-in-difference, 0.40 [95% CI, 0.14 to 0.67] deaths by suicide per 100 000 population), October (1.28 vs 0.75 deaths by suicide per 100 000 population; difference-in-difference, 0.62 [95% CI, 0.48 to 0.77] deaths by suicide per 100 000 population) and November (0.96 vs 0.76 deaths by suicide per 100 000 population; difference-in-difference, 0.29 [95% CI, 0.15 to 0.44] deaths by suicide per 100 000 population). Among women aged younger than 30 years, suicide rates in 2020 compared with 2016 to 2019 were decreased in April (0.32 vs 0.43 deaths by suicide per 100 000 population; difference-in-difference, −0.18 [95% CI, −0.31 to −0.04] deaths by suicide per 100 000 population) and increased in August through November, with difference-in-difference estimates ranging from 0.16 (95% CI, 0.03 to 0.30) deaths by suicide per 100 000 population in November to 0.29 (95% CI, 0.16 to 0.43) in October. Suicide rates were also increased in July through November among women aged 30 to 49 years, with difference-in-difference estimates ranging from 0.24 (95% CI, 0.04 to 0.44) deaths by suicide per 100 000 population in July to 0.89 (95% CI, 0.68 to 1.09) in October, and among women aged 50 to 69 years, with difference-in-difference estimates ranging from 0.33 (95% CI, 0.12 to 0.55) deaths by suicide per 100 000 population in July, August, and September to 0.69 (95% CI, 0.48 to 0.91) in October. Suicide rates among women aged 70 years or older were increased in November (difference-in-difference, 0.64 [95% CI, 0.27 to 1.00] deaths by suicide per 100 000 population).

**Table 2.** Individuals Who Died of Suicide, Suicide Rates, and Difference-in-Difference Analysis of the Suicide Rates in April Through November 2020 vs 2016-2019 in Women

Age group	Deaths, No. (rate per 100 000 population)^a^										
	Jan	Feb	Ma	Apr	May	Jun	Jul	Aug	Sep	Oct	Nov
**Total**											
2016-2019	497 (0.77)	481 (0.74)	545 (0.84)	541 (0.83)	570 (0.88)	517 (0.80)	532 (0.82)	502 (0.77)	504 (0.78)	486 (0.75)	493 (0.76)
2020	462 (0.71)	392 (0.61)	480 (0.74)	410 (0.63)	455 (0.70)	483 (0.75)	623 (0.96)	635 (0.98)	629 (0.97)	826 (1.28)	620 (0.96)
Difference-in-difference, deaths per 100 000 population (95% CI)^b^	NA	NA	NA	−0.10 (−0.25 to 0.04)	−0.08 (−0.22 to 0.06)	0.04 (−0.10 to 0.19)	0.24 (0.09 to 0.38)	0.3 (0.16 to 0.45)	0.29 (0.15 to 0.44)	0.62 (0.48 to 0.77)	0.29 (0.15 to 0.44)
**<30 y**											
2016-2019	60 (0.36)	62 (0.37)	68 (0.41)	71 (0.43)	65 (0.39)	57 (0.34)	70 (0.42)	63 (0.38)	64 (0.39)	63 (0.38)	57 (0.35)
2020	70 (0.43)	75 (0.46)	75 (0.46)	52 (0.32)	58 (0.36)	79 (0.48)	96 (0.59)	118 (0.72)	113 (0.69)	121 (0.74)	94 (0.58)
Difference-in-difference, deaths per 100 000 population (95% CI)^b^	NA	NA	NA	−0.18 (−0.31 to −0.04)	−0.10 (−0.24 to 0.03)	0.07 (−0.06 to 0.21)	0.10 (−0.04 to 0.23)	0.28 (0.14 to 0.41)	0.24 (0.11 to 0.37)	0.29 (0.16 to 0.43)	0.16 (0.03 to 0.30)
**30-49 y**											
2016-2019	131 (0.79)	129 (0.78)	138 (0.83)	129 (0.78)	142 (0.85)	137 (0.83)	127 (0.77)	137 (0.83)	135 (0.82)	126 (0.76)	122 (0.74)
2020	116 (0.73)	95 (0.59)	124 (0.78)	99 (0.62)	108 (0.68)	123 (0.77)	145 (0.91)	171 (1.07)	178 (1.11)	247 (1.54)	180 (1.13)
Difference-in-difference, deaths per 100 000 population (95% CI)^b^	NA	NA	NA	−0.06 (−0.26 to 0.14)	−0.08 (−0.28 to 0.12)	0.04 (−0.16 to 0.24)	0.24 (0.04 to 0.44)	0.34 (0.14 to 0.55)	0.40 (0.20 to 0.60)	0.89 (0.68 to 1.09)	0.49 (0.29 to 0.69)
**50-69 y**											
2016-2019	153 (0.91)	148 (0.88)	167 (0.99)	165 (0.98)	181 (1.08)	149 (0.89)	167 (0.99)	146 (0.87)	145 (0.86)	148 (0.88)	151 (0.90)
2020	132 (0.81)	99 (0.61)	148 (0.91)	126 (0.77)	131 (0.80)	127 (0.78)	191 (1.17)	171 (1.05)	170 (1.04)	232 (1.42)	179 (1.10)
Difference-in-difference, deaths per 100 000 population (95% CI)^b^	NA	NA	NA	−0.06 (−0.27 to 0.16)	−0.12 (−0.34 to 0.09)	0.05 (−0.17 to 0.26)	0.33 (0.12 to 0.55)	0.33 (0.12 to 0.55)	0.33 (0.12 to 0.55)	0.69 (0.48 to 0.91)	0.36 (0.14 to 0.57)
**≥70 y**											
2016-2019	153 (1.01)	141 (0.93)	172 (1.14)	175 (1.16)	181 (1.20)	173 (1.15)	167 (1.11)	155 (1.03)	159 (1.06)	148 (0.98)	163 (1.08)
2020	144 (0.90)	122 (0.76)	130 (0.81)	133 (0.83)	157 (0.98)	154 (0.96)	191 (1.19)	175 (1.09)	168 (1.05)	226 (1.41)	164 (1.02)
Difference-in-difference, deaths per 100 000 population (95% CI)^b^	NA	NA	NA	−0.12 (−0.48 to 0.24)	−0.01 (−0.38 to 0.35)	0.02 (−0.35 to 0.38)	0.29 (−0.07 to 0.65)	0.27 (−0.09 to 0.63)	0.20 (−0.16 to 0.57)	0.64 (0.27 to 1.00)	0.15 (−0.21 to 0.51)

Abbreviation: NA, not applicable.

^a^ Excluding 116 women who died of suicide for whom information about age was not available.

^b^ Subtraction of differences in January through April from differences in April through September. Negative values represent lower suicide rates in 2020 vs 2016 to 2019.

The results of the analyses by occupational status are shown in eTable 1 in the Supplement for men and in eTable 2 in the Supplement for women. Significant increases in the number of men who died of suicide were observed in August through October for employed men and in August, September, and November among students. Significant increases in the number of women who died of suicide were observed in July, September, October, and November for employed women, in August and September for students, in August, October, and November among homemakers, and in July through October for women who were unemployed.

### Analysis of Observed vs Expected Suicide Rates

**Figure 2.**
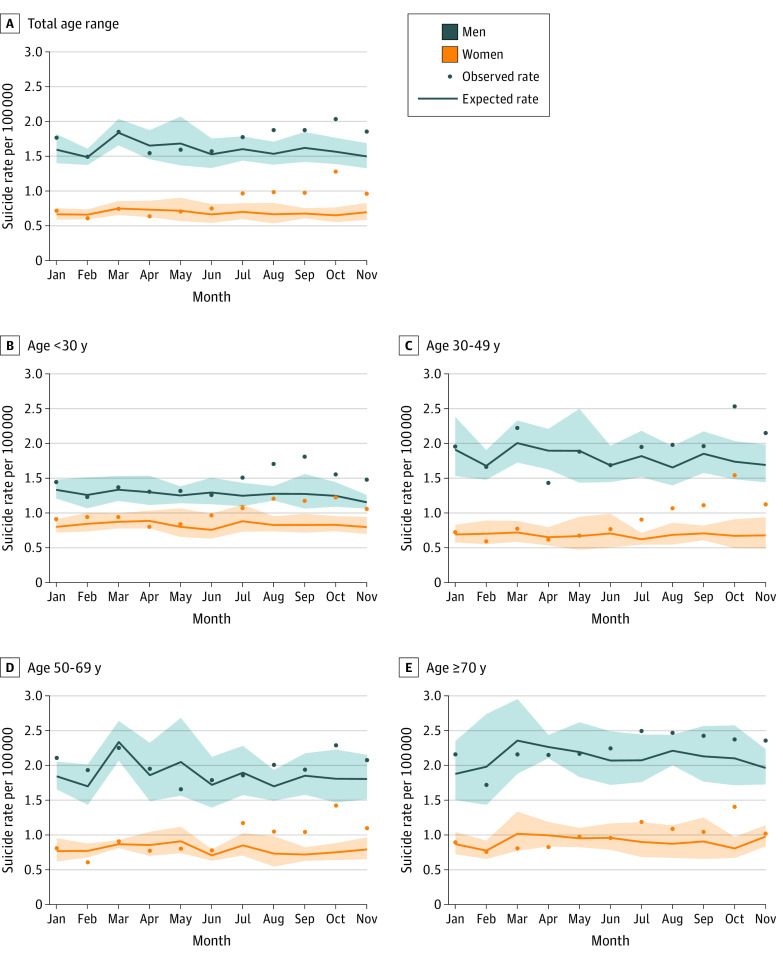
Observed and Expected Monthly Suicide Rates in Japan from January to November 2020 by Age Shaded areas indicate 95% CIs for expected suicide rates.

Analyses of observed vs expected suicide rates were based on 142 330 men and 66 149 women (31.7% of total) who died by suicide. Between 2011 and 2019, suicide rates decreased in all 11 investigated months. (eTable 3, eTable 4, and eFigures 2-6 in the Supplement). Figure 2 shows the observed vs expected monthly rates in 2020. The observed vs expected suicide rate for men in 2020 was higher in January (1.77 vs 1.59 deaths by suicide per 100 000 population; RR, 1.11 [95% CI, 1.04-1.17]), lower in April (1.54 vs 1.65 deaths by suicide per 100 000 population; RR, 0.93 [95% CI, 0.88 to 0.99]), and higher in July (1.77 vs 1.60 deaths by suicide per 100 000 population; RR, 1.11 [95% CI, 1.04 to 1.17]), August (1.87 vs 1.53 deaths by suicide per 100 000 population; RR, 1.22 [95% CI, 1.15 to 1.29]), September (1.88 vs 1.62 deaths by suicide per 100 000 population; RR, 1.16 [95% CI, 1.09 to 1.23]), October (2.03 vs 1.56 deaths by suicide per 100 000 population; RR, 1.30 [95% CI, 1.23 to 1.37]), and November (1.85 vs 1.50 deaths by suicide per 100 000 population; RR, 1.24 [95% CI, 1.17 to 1.31]) (eTable 3 in the Supplement). The largest ratios of the observed vs expected suicide rate were observed among individuals aged younger than 30 years, with the RRs ranging from 1.34 (95% CI, 1.14 to 1.54) in July to 1.68 (95% CI, 1.46 to 1.89) in September. The lower-than-expected suicide rate in April was associated with men aged 30 to 49 (1.43 vs 1.90 deaths by suicide per 100 000 population; RR, 0.76 [95% CI, 0.66-0.85]).

The observed vs expected suicide rate for women in 2020 was lower in April (0.63 vs 0.73 deaths by suicide per 100 000 population; RR, 0.87 [95% CI, 0.78 to 0.95]) and higher in June (0.75 vs 0.66 deaths by suicide per 100 000 population; RR, 1.13 [95% CI, 1.03 to 1.23]), July (0.96 vs 0.70 deaths by suicide per 100 000 population; RR, 1.38 [95% CI, 1.27 to 1.49]), August (0.98 vs 0.66 deaths by suicide per 100 000 population; RR, 1.48 [95% CI, 1.36 to 1.60]), September (0.97 vs 0.67 deaths by suicide per 100 000 population; RR, 1.44 [95% CI, 1.33 to 1.56]), October (1.28 vs 0.65 deaths by suicide per 100 000 population; RR, 1.97 [95% CI, 1.84 to 2.11]), and November (0.96 vs 0.69 deaths by suicide per 100 000 population; RR, 1.38 [95% CI, 1.27 to 1.49]) (eTable 4 in the Supplement). The largest ratios of the observed vs expected suicide rates were observed among women aged younger than 30 years (observed vs expected suicide rate in October: 0.74 vs 0.35 suicide deaths per 100 000 population; RR, 2.14 [95% CI, 1.76 to 2.52]) and those aged 30 to 49 years (observed vs expected suicide rate in October: 1.54 vs 0.67 deaths by suicide per 100 0000 population; RR, 2.30 [95% CI, 2.01 to 2.58]).

## Discussion

This cross-sectional study found that suicide rates in Japan were increased among men in October and November 2020 and among women in July through November 2020 compared with the corresponding months in 2016 to 2019. Among men aged 30 years or younger, suicide rates were increased in July through November; the relative increase was most pronounced in this age group. Similarly, among women, the largest relative increase was observed among those aged younger than 30 years and 30 to 49 years.

A previous study from Japan^5^ using data through September 2020 found an excess suicide mortality in July through September among women but not men. Our analyses expand on the knowledge regarding suicide rates in Japan during the COVID-19 pandemic by using data on suicide mortality through November 2020, by providing analyses by age group and occupational status, and by using 2 different methods for assessing changes in suicide rates vs previous years (a difference-in-difference method and a comparison of the observed vs expected suicide rates based on trends from 9 previous years).

Although suicides may have been influenced by factors specific to 2020 but not associated with the COVID-19 pandemic, our findings align with observations that young adults and women experienced disproportionate mental health^11^ or suicidal ideation^12^ burdens during the COVID-19 pandemic. In the context of Japan, it has been suggested that women and younger adults are particularly susceptible to the economic downturn after social distancing measures introduced to curb the pandemic. Women and younger adults are overrepresented among individuals with irregular employment and workers in service industries, including restaurants and tourism, which have been severely affected by the pandemic. In July through September 2020, the decrease in the number of working individuals compared with the previous year was around 2-fold larger for women than men.^13^ However, by October, the decrease in working individuals among men and women had become similar (460 000 fewer men and 470 000 fewer women working compared with the previous year).^13^ Prior research has demonstrated an association between unemployment and increased rates of suicide in Japan,^14,15^ raising concerns that the financial stress and unemployment associated with the pandemic have contributed to economic drivers of suicide.^1,2,3^

### Limitations

Our study has limitations. Suicide rates may have been influenced by factors unrelated to the pandemic, and the observed changes in monthly suicide rates may partly reflect random fluctuations. Importantly, while we assessed whether suicide rates were increased starting from April 2020, the increase in suicide rates occurred later in the year for both men and women. These findings were not included in the prespecified hypothesis and should be interpreted with caution. Moreover, we assessed suicide rates in each month separately and did not examine whether the total number of individuals who died of suicide increased during the pandemic. In line with evidence from previous epidemics indicating that a short-term decrease in suicide rates can occur initially,^16^ suicide rates were decreased in April among women aged younger than 30 years and men aged 30 to 49 years and in May among men aged 50 to 69 years. Future studies are needed to examine the overall association of the pandemic with suicide rates. Additionally, owing to the lack of longitudinal data on the individual level, we could not examine the association between changes in occupational status in association with the pandemic and risk of suicide.

## Conclusions

This cross-sectional study found that compared with previous years, suicide rates in 2020 in Japan had increased in October and November for men and in July through November for women. The relative increases were particularly pronounced among men aged younger than 30 years and women aged younger than 30 years and 30 to 49 years.

## Notes

Supplement. **eTable 1.** Difference-in-Difference Model Assessing Suicides in April Through September 2020 vs 2016 to 2019 in Men by Occupational Status Group **eTable 2.** Difference-in-Difference Model Assessing Suicides in April Through September 2020 vs 2016 to 2019 in Women by Occupational Status Group **eTable 3.** Observed and Expected Monthly Suicide Rates Among Men in 2020 Based on Trends From 2011 to 2019 Stratified by Age **eTable 4.** Observed and Expected Monthly Suicide Rates Among Women in 2020 Based on Trends From 2011 to 2019 Stratified by Age **eFigure 1.** Observed and Expected Monthly Number of Individuals Who Died of Suicide in January Through November, 2011 to 2020, by Occupational Status Category **eFigure 2.** Observed and Expected Monthly Suicide Rates in January to November, 2011 to 2020, in All Ages **eFigure 3.** Observed and Expected Monthly Suicide Rates in January to November, 2011 to 2020, Among Individuals Aged Younger Than 30 Years **eFigure 4.** Observed and Expected Monthly Suicide Rates in January to November, 2011 to 2020, Among Individuals Aged 30 to 49 Years **eFigure 5.** Observed and Expected Monthly Suicide Rates in January to November, 2011 to 2020, Among Individuals Aged 50 to 69 Years **eFigure 6.** Observed and Expected Monthly Suicide Rates in January to November, 2011 to 2020, Among Individuals Aged 70 Years or Older
