# Supplementary material for: Assessment of Suicide in Japan During the COVID-19 Pandemic vs Previous Years
Source: JAMA Netw Open. 2021 Feb 2;4(2):e2037378. doi: 10.1001/jamanetworkopen.2020.37378 (PMC7856546; doi:10.1001/jamanetworkopen.2020.37378)
Supplement: Supplement. — eTable 1. Difference-in-Difference Model Assessing Suicides in April Through September 2020 vs 2016 to 2019 in Men by Occupational Status Group eTable 2. Difference-in-Difference Model Assessing Suicides in April Through September 2020 vs 2016 to 2019 in Women by Occupational Status Group eTable 3. Observed and Expected Monthly Suicide Rates Among Men in 2020 Based on Trends From 2011 to 2019 Stratified by Age eTable 4. Observed and Expected Monthly Suicide Rates Among Women in 2020 Based on Trends From 2011 to 2019 Stratified by Age eFigure 1. Observed and Expected Monthly Number of Individuals Who Died of Suicide in January Through November, 2011 to 2020, by Occupational Status Category eFigure 2. Observed and Expected Monthly Suicide Rates in January to November, 2011 to 2020, in All Ages eFigure 3. Observed and Expected Monthly Suicide Rates in January to November, 2011 to 2020, Among Individuals Aged Younger Than 30 Years eFigure 4. Observed and Expected Monthly Suicide Rates in January to November, 2011 to 2020, Among Individuals Aged 30 to 49 Years eFigure 5. Observed and Expected Monthly Suicide Rates in January to November, 2011 to 2020, Among Individuals Aged 50 to 69 Years eFigure 6. Observed and Expected Monthly Suicide Rates in January to November, 2011 to 2020, Among Individuals Aged 70 Years or Older [file jamanetwopen-e2037378-s001.pdf]

Supplemental Online Content

Sakamoto H, Ishikane M, Ghaznavi C, Ueda P. Assessment of suicide in Japan during the COVID-19 pandemic vs previous years. *JAMA Netw Open*. 2021;4(2):e2037378. doi:10.1001/jamanetworkopen.2020.37378

eTable 1. Difference-in-Difference Model Assessing Suicides in April Through September 2020 vs 2016 to 2019 in Men by Occupational Status Group
eTable 2. Difference-in-Difference Model Assessing Suicides in April Through September 2020 vs 2016 to 2019 in Women by Occupational Status Group
eTable 3. Observed and Expected Monthly Suicide Rates Among Men in 2020 Based on Trends From 2011 to 2019 Stratified by Age
eTable 4. Observed and Expected Monthly Suicide Rates Among Women in 2020 Based on Trends From 2011 to 2019 Stratified by Age
eFigure 1. Observed and Expected Monthly Number of Individuals Who Died of Suicide in January Through November, 2011 to 2020, by Occupational Status Category
eFigure 2. Observed and Expected Monthly Suicide Rates in January to November, 2011 to 2020, in All Ages
eFigure 3. Observed and Expected Monthly Suicide Rates in January to November, 2011 to 2020, Among Individuals Aged Younger Than 30 Years
eFigure 4. Observed and Expected Monthly Suicide Rates in January to November, 2011 to 2020, Among Individuals Aged 30 to 49 Years
eFigure 5. Observed and Expected Monthly Suicide Rates in January to November, 2011 to 2020, Among Individuals Aged 50 to 69 Years
eFigure 6. Observed and Expected Monthly Suicide Rates in January to November, 2011 to 2020, Among Individuals Aged 70 Years or Older

This supplemental material has been provided by the authors to give readers additional information about their work.

© 2021 Sakamoto H et al. *JAMA Network Open.*

eTable 1. Difference-in-Difference Model Assessing Suicides in April Through September 2020 vs 2016 to 2019 in Men by Occupational Status Group

	January	February	March	April	May	June	July	August	September	October	November
**Family business/self-employed**											
2016-2019, mean n	98	93	110	98	106	96	88	86	94	99	87
2020, n	99	95	96	79	72	76	66	80	70	79	74
Difference-in-difference (95% CI)[a]				-15 (-44 to 14)	-31 (-59 to -2)	-16 (-45 to 12)	-18 (-47 to 11)	-2 (-31 to 26)	-20 (-49 to 9)	-16 (-46 to 14)	-10 (-39 to 20)
**Employee**											
2016-2019, mean n	414	353	424	382	407	367	371	353	369	369	342
2020, n	405	314	408	291	333	287	365	396	410	475	384
Difference-in-difference (95% CI)[a]				-70 (-128 to 12)	-53 (-111 to 5)	-59 (-117 to 1)	15 (-43 to 73)	64 (6 to 123)	62 (4 to 120)	128 (65 to 191)	63 (0 to 126)
**Student**											
2016-2019, mean n	85	81	91	90	70	69	77	75	94	86	68
2020, n	80	62	80	62	84	78	90	128	138	94	116
Difference-in-difference (95% CI)[a]				-16 (-54 to 22)	26 (-12 to 64)	21 (-18 to 59)	25 (-14 to 63)	65 (27 to 103)	56 (17 to 94)	20 (-17 to 57)	60 (23 to 97)
**Unemployed**											
2016-2019, mean n	479	464	584	539	574	518	538	497	487	490	482
2020, n	454	401	505	456	445	454	496	487	473	501	513
Difference-in-difference (95% CI)[a]				-28 (-133 to 77)	-73 (-179 to 32)	-8 (-114 to 97)	14 (-92 to 119)	45 (-60 to 151)	41 (-64 to 147)	66 (-40 to 173)	87 (-20 to 193)
N=2726 suicides with missing data on occupational status were not included in the analyses.<br>[a] Subtraction of differences in January through April from differences in April through September. Negative values represent fewer suicides in 2020 vs 2016-2019.											

© 2021 Sakamoto H et al. *JAMA Network Open.*

eTable 2. Difference-in-Difference Model Assessing Suicides in April Through September 2020 vs 2016 to 2019 in Women by Occupational Status Group

	January	February	March	April	May	June	July	August	September	October	November
**Family business/self-employed**											
2016-2019, mean n	12	13	14	16	13	13	12	11	13	12	13
2020, n	14	9	10	8	9	14	12	7	16	16	18
Difference-in-difference (95% CI)[a]				-6 (-15 to 3)	-2 (-11 to 7)	3 (-6 to 12)	2 (-7 to 11)	-2 (-11 to 7)	5 (-4 to 14)	6 (-2 to 15)	7 (-2 to 15)
**Employee**											
2016-2019, mean n	80	83	85	81	93	72	85	90	81	81	82
2020, n	93	65	92	63	93	86	124	118	123	175	131
Difference-in-difference (95% CI)[a]				-19 (-53 to 16)	-1 (-36 to 34)	13 (-21 to 48)	38 (3 to 73)	27 (-8 to 62)	41 (6 to 76)	93 (60 to 125)	48 (15 to 81)
**Student**											
2016-2019, mean n	39	34	38	41	37	33	35	35	33	36	29
2020, n	60	48	38	28	30	42	40	96	70	54	58
Difference-in-difference (95% CI)[a]				-25 (-48 to -1)	-18 (-42 to 5)	-3 (-26 to 21)	-7 (-30 to 17)	50 (26 to 73)	25 (2 to 49)	6 (-18 to 31)	17 (-7 to 42)
**Homemaker**											
2016-2019, mean n	89	77	86	91	98	91	96	80	81	79	79
2020, n	54	59	71	73	77	69	105	105	89	141	95
Difference-in-difference (95% CI)[a]				4 (-31 to 39)	2 (-34 to 37)	1 (-35 to 36)	32 (-3 to 67)	47 (12 to 82)	31 (-5 to 66)	85 (50 to 120)	38 (4 to 73)
**Unemployed**											
2016-2019, mean n	264	248	295	284	299	281	272	252	260	248	265
2020, n	235	207	242	221	226	244	302	285	302	368	278
Difference-in-difference (95% CI)[a]				-22 (-82 to 37)	-32 (-92 to 27)	4 (-56 to 63)	70 (11 to 130)	74 (14 to 134)	83 (23 to 142)	161 (100 to 223)	54 (-8 to 115)
N=1225 suicides with missing data on occupational status were not included in the analyses.											

© 2021 Sakamoto H et al. *JAMA Network Open.*

[a] Subtraction of differences in January through April from differences in April through September. Negative values represent fewer suicides in 2020 vs 2016-2019.				

© 2021 Sakamoto H et al. *JAMA Network Open.*

eTable 3. Observed and Expected Monthly Suicide Rates Among Men in 2020 Based on Trends From 2011 to 2019 Stratified by Age

	January	February	March	April	May	June	July	August	September	October	November
**Total**											
Yearly change 2011-2019, % (95% CI)	-5.5 (-7.1 to -3.8)	-5.1 (-6.1 to -4.1)	-4.7 (-6 to -3.4)	-5.7 (-7.3 to -4.1)	-6.7 (-9.3 to -4.1)	-6.5 (-8.3 to -4.8)	-5.4 (-6.8 to -4.1)	-5.5 (-6.9 to -4.2)	-4.9 (-6.6 to -3.2)	-5.6 (-7.1 to -4.1)	-5 (-6.5 to -3.5)
Observed rate in 2020	1.77	1.49	1.85	1.54	1.59	1.57	1.77	1.87	1.88	2.03	1.85
Expected rate in 2020 (95% CI)	1.59 (1.4 - 1.82)	1.49 (1.37 - 1.61)	1.84 (1.66 - 2.04)	1.65 (1.46 - 1.87)	1.68 (1.37 - 2.07)	1.53 (1.33 - 1.75)	1.6 (1.44 - 1.79)	1.53 (1.38 - 1.71)	1.62 (1.42 - 1.85)	1.56 (1.39 - 1.76)	1.5 (1.33 - 1.69)
Observed vs expected rate ratio (95% CI)	1.11 (1.04 - 1.17)	1 (0.94 - 1.07)	1.01 (0.95 - 1.07)	0.93 (0.88 - 0.99)	0.95 (0.89 - 1.01)	1.03 (0.96 - 1.09)	1.11 (1.04 - 1.17)	1.22 (1.15 - 1.29)	1.16 (1.09 - 1.23)	1.3 (1.23 - 1.37)	1.24 (1.17 - 1.31)
**<30 y**											
Yearly change 2011-2019, % (95% CI)	-2.8 (-4.8 to -0.8)	-2.3 (-5.9 to 1.2)	-4.6 (-7.2 to -2)	-4.8 (-8 to -1.6)	-6.2 (-8.2 to -4.2)	-3.6 (-6.5 to -0.6)	-4.2 (-7 to -1.5)	-3.3 (-4.9 to -1.7)	-3.8 (-7.7 to 0.1)	-3.8 (-6.6 to -0.9)	-4.6 (-6.4 to -2.8)
Observed rate in 2020	0.96	0.75	0.89	0.82	0.84	0.78	1.03	1.22	1.33	1.07	1
Expected rate in 2020 (95% CI)	0.85 (0.73 - 1)	0.78 (0.59 - 1.03)	0.85 (0.69 - 1.05)	0.82 (0.63 - 1.06)	0.77 (0.66 - 0.9)	0.81 (0.64 - 1.03)	0.77 (0.62 - 0.95)	0.79 (0.7 - 0.9)	0.79 (0.58 - 1.08)	0.77 (0.61 - 0.96)	0.67 (0.58 - 0.78)
Observed vs expected rate ratio (95% CI)	1.13 (0.96 - 1.3)	0.96 (0.8 - 1.13)	1.04 (0.88 - 1.21)	1.01 (0.84 - 1.17)	1.08 (0.91 - 1.26)	0.96 (0.79 - 1.12)	1.34 (1.14 - 1.54)	1.54 (1.33 - 1.75)	1.68 (1.46 - 1.89)	1.4 (1.2 - 1.61)	1.48 (1.26 - 1.71)
**30-49 y**											

© 2021 Sakamoto H et al. *JAMA Network Open.*

Yearly change 2011-2019, % (95% CI)	-5.3 (-8 to -2.5)	-5.4 (-7 to -3.8)	-5.2 (-7.1 to -3.3)	-5 (-7 to -3.1)	-6.4 (-9.9 to -2.9)	-6 (-8 to -4.1)	-4.2 (-6.5 to -1.9)	-6.4 (-8.5 to -4.2)	-3.5 (-5.5 to -1.4)	-5.7 (-7.7 to -3.8)	-4.8 (-6.8 to -2.8)
Observed rate in 2020	1.96	1.67	2.22	1.43	1.88	1.69	1.95	1.98	1.96	2.53	2.15
Expected rate in 2020 (95% CI)	1.91 (1.53 - 2.39)	1.68 (1.48 - 1.91)	2.01 (1.73 - 2.33)	1.9 (1.63 - 2.21)	1.9 (1.44 - 2.5)	1.69 (1.45 - 1.97)	1.82 (1.52 - 2.18)	1.66 (1.4 - 1.96)	1.85 (1.58 - 2.18)	1.74 (1.49 - 2.04)	1.69 (1.44 - 1.98)
Observed vs expected rate ratio (95% CI)	1.02 (0.91 - 1.14)	0.99 (0.87 - 1.11)	1.11 (0.99 - 1.22)	0.76 (0.66 - 0.85)	0.99 (0.88 - 1.1)	1 (0.88 - 1.12)	1.07 (0.95 - 1.19)	1.2 (1.07 - 1.33)	1.06 (0.94 - 1.17)	1.46 (1.32 - 1.6)	1.27 (1.14 - 1.4)
**50-69 y**											
Yearly change 2011-2019, % (95% CI)	-7.6 (-9 to -6.3)	-7.4 (-9.6 to -5.3)	-5.4 (-6.9 to -3.9)	-8.1 (-10.9 to -5.3)	-7.6 (-11 to -4.2)	-8.6 (-11.2 to -5.9)	-6.7 (-9 to -4.3)	-6.9 (-8.6 to -5.3)	-6.4 (-8.5 to -4.4)	-7 (-9.6 to -4.3)	-5.6 (-7.8 to -3.4)
Observed rate in 2020	2.11	1.94	2.25	1.95	1.66	1.79	1.86	2.01	1.94	2.29	2.08
Expected rate in 2020 (95% CI)	1.85 (1.66 - 2.06)	1.7 (1.44 - 2.02)	2.34 (2.07 - 2.64)	1.86 (1.49 - 2.33)	2.05 (1.57 - 2.69)	1.72 (1.4 - 2.13)	1.9 (1.58 - 2.28)	1.7 (1.49 - 1.94)	1.85 (1.58 - 2.18)	1.81 (1.47 - 2.23)	1.81 (1.52 - 2.15)
Observed vs expected rate ratio (95% CI)	1.14 (1.02 - 1.27)	1.14 (1.01 - 1.26)	0.96 (0.86 - 1.06)	1.05 (0.93 - 1.17)	0.81 (0.71 - 0.91)	1.04 (0.92 - 1.16)	0.98 (0.87 - 1.09)	1.18 (1.05 - 1.31)	1.05 (0.93 - 1.16)	1.27 (1.14 - 1.4)	1.15 (1.03 - 1.27)
**≥70 y**											
Yearly change 2011-	-4.3 (-7.1 to -1.5)	-2.9 (-7 to 1.2)	-3 (-5.9 to -0.2)	-4 (-4.9 to -3.1)	-6.5 (-8.7 to -4.3)	-6.8 (-9.1 to -4.5)	-6.8 (-8.8 to -4.7)	-4.9 (-6.1 to -3.6)	-6.2 (-8.6 to -3.9)	-5.7 (-8.2 to -3.1)	-5.8 (-7.4 to -4.2)

© 2021 Sakamoto H et al. *JAMA Network Open.*

2019, % (95% CI)											
Observed rate in 2020	2.16	1.72	2.16	2.15	2.17	2.25	2.5	2.47	2.43	2.38	2.36
Expected rate in 2020 (95% CI)	1.88 (1.51 - 2.35)	1.98 (1.44 - 2.74)	2.36 (1.88 - 2.95)	2.27 (2.11 - 2.44)	2.19 (1.84 - 2.62)	2.07 (1.72 - 2.49)	2.08 (1.76 - 2.44)	2.21 (2.01 - 2.44)	2.13 (1.77 - 2.57)	2.1 (1.72 - 2.58)	1.97 (1.73 - 2.23)
Observed vs expected rate ratio (95% CI)	1.15 (1.01 - 1.29)	0.87 (0.75 - 0.99)	0.92 (0.8 - 1.03)	0.95 (0.83 - 1.07)	0.99 (0.87 - 1.11)	1.08 (0.95 - 1.22)	1.2 (1.06 - 1.34)	1.12 (0.99 - 1.25)	1.14 (1.01 - 1.27)	1.13 (1 - 1.26)	1.2 (1.06 - 1.34)
Rates are shown in suicides per 100,000 person-years.<br>N = 602 suicides with missing data on age were not included in the age-specific analyses.											

© 2021 Sakamoto H et al. *JAMA Network Open.*

eTable 4. Observed and Expected Monthly Suicide Rates Among Women in 2020 Based on Trends From 2011 to 2019 Stratified by Age

	January	February	March	April	May	June	July	August	September	October	November
**Total**											
Yearly change 2011-2019, % (95% CI)	-5.7 (-7.3 to -4.2)	-4.3 (-5.6 to -2.9)	-4.9 (-6.6 to -3.2)	-5.1 (-7.1 to -3)	-7.2 (-10.2 to -4.3)	-6.8 (-9.4 to -4.3)	-6.4 (-8.5 to -4.4)	-6.5 (-9.3 to -3.7)	-6.1 (-7.5 to -4.7)	-6.2 (-8.2 to -4.2)	-4.4 (-6.6 to -2.2)
Observed rate in 2020	0.71	0.61	0.74	0.63	0.7	0.75	0.96	0.98	0.97	1.28	0.96
Expected rate in 2020 (95% CI)	0.66 (0.59 - 0.75)	0.66 (0.59 - 0.73)	0.75 (0.65 - 0.85)	0.73 (0.62 - 0.86)	0.71 (0.57 - 0.9)	0.66 (0.54 - 0.81)	0.7 (0.59 - 0.82)	0.66 (0.53 - 0.83)	0.67 (0.6 - 0.75)	0.65 (0.55 - 0.76)	0.69 (0.58 - 0.83)
Observed vs expected rate ratio (95% CI)	1.08 (0.98 - 1.18)	0.92 (0.83 - 1.01)	0.99 (0.9 - 1.08)	0.87 (0.78 - 0.95)	0.99 (0.89 - 1.08)	1.13 (1.03 - 1.23)	1.38 (1.27 - 1.49)	1.48 (1.36 - 1.6)	1.44 (1.33 - 1.56)	1.97 (1.84 - 2.11)	1.38 (1.27 - 1.49)
**<30 y**											
Yearly change 2011-2019, % (95% CI)	-4.7 (-8.4 to -1)	-0.2 (-4.8 to 4.4)	-2.7 (-6.2 to 0.8)	-1.8 (-5.7 to 2.1)	-6.8 (-14.5 to 0.8)	-8.2 (-15.9 to -0.5)	-1.4 (-7.3 to 4.5)	-3.5 (-7.4 to 0.4)	-4.8 (-9.6 to 0)	-3 (-6.9 to 0.8)	-4 (-8.8 to 0.8)
Observed rate in 2020	0.43	0.46	0.46	0.32	0.36	0.48	0.59	0.72	0.69	0.74	0.58
Expected rate in 2020 (95% CI)	0.32 (0.24 - 0.43)	0.36 (0.25 - 0.52)	0.39 (0.3 - 0.51)	0.4 (0.3 - 0.55)	0.32 (0.17 - 0.58)	0.28 (0.15 - 0.51)	0.4 (0.25 - 0.64)	0.35 (0.25 - 0.47)	0.35 (0.24 - 0.51)	0.35 (0.25 - 0.47)	0.31 (0.22 - 0.46)
Observed vs expected rate ratio (95% CI)	1.35 (1.04 - 1.67)	1.27 (0.98 - 1.56)	1.18 (0.91 - 1.44)	0.79 (0.57 - 1)	1.12 (0.83 - 1.41)	1.76 (1.37 - 2.15)	1.48 (1.18 - 1.77)	2.1 (1.72 - 2.47)	2.01 (1.64 - 2.38)	2.14 (1.76 - 2.52)	1.83 (1.46 - 2.2)

© 2021 Sakamoto H et al. *JAMA Network Open.*

**30-49 y**											
Yearly change 2011-2019, % (95% CI)	-5.7 (-8 to -3.4)	-3.4 (-6.4 to -0.4)	-6 (-8.6 to -3.3)	-6.6 (-9.1 to -4.1)	-8.5 (-12.8 to -4.1)	-5.9 (-10.1 to -1.7)	-8.5 (-10.2 to -6.8)	-7.1 (-9.9 to -4.2)	-5.5 (-7.4 to -3.7)	-5.8 (-9.6 to -2)	-4.9 (-9 to -0.8)
Observed rate in 2020	0.73	0.59	0.78	0.62	0.68	0.77	0.91	1.07	1.11	1.54	1.13
Expected rate in 2020 (95% CI)	0.69 (0.58 - 0.83)	0.7 (0.55 - 0.89)	0.72 (0.59 - 0.89)	0.65 (0.54 - 0.8)	0.67 (0.47 - 0.95)	0.71 (0.51 - 0.99)	0.62 (0.55 - 0.71)	0.69 (0.55 - 0.86)	0.71 (0.61 - 0.82)	0.67 (0.5 - 0.91)	0.68 (0.49 - 0.94)
Observed vs expected rate ratio (95% CI)	1.05 (0.86 - 1.24)	0.84 (0.67 - 1.01)	1.08 (0.89 - 1.26)	0.95 (0.76 - 1.13)	1.01 (0.82 - 1.2)	1.09 (0.9 - 1.28)	1.45 (1.22 - 1.69)	1.56 (1.32 - 1.79)	1.57 (1.34 - 1.8)	2.3 (2.01 - 2.58)	1.66 (1.41 - 1.9)
**50-69 y**											
Yearly change 2011-2019, % (95% CI)	-6.6 (-9.3 to -3.9)	-5.5 (-7.1 to -3.8)	-5.5 (-6.4 to -4.7)	-5.1 (-7.6 to -2.6)	-5.8 (-8.4 to -3.2)	-8.2 (-9.6 to -6.7)	-6.3 (-8.7 to -4)	-7.2 (-10.9 to -3.5)	-6.7 (-8.4 to -5)	-6.7 (-8.7 to -4.7)	-5 (-7.5 to -2.5)
Observed rate in 2020	0.81	0.61	0.91	0.77	0.8	0.78	1.17	1.05	1.04	1.42	1.1
Expected rate in 2020 (95% CI)	0.77 (0.62 - 0.95)	0.77 (0.68 - 0.88)	0.87 (0.81 - 0.93)	0.86 (0.7 - 1.05)	0.91 (0.74 - 1.12)	0.71 (0.63 - 0.79)	0.85 (0.71 - 1.03)	0.73 (0.55 - 0.99)	0.72 (0.63 - 0.82)	0.75 (0.64 - 0.88)	0.79 (0.65 - 0.97)
Observed vs expected rate ratio (95% CI)	1.05 (0.87 - 1.23)	0.79 (0.63 - 0.94)	1.05 (0.88 - 1.21)	0.9 (0.75 - 1.06)	0.88 (0.73 - 1.03)	1.1 (0.91 - 1.29)	1.38 (1.18 - 1.57)	1.43 (1.22 - 1.65)	1.45 (1.23 - 1.67)	1.89 (1.65 - 2.14)	1.39 (1.18 - 1.59)
**≥70 y**											

© 2021 Sakamoto H et al. *JAMA Network Open.*

Yearly change 2011-2019, % (95% CI)	-5.9 (-8.2 to -3.6)	-6.5 (-8.6 to -4.4)	-4.8 (-8.3 to -1.4)	-6.2 (-8.5 to -4)	-8.5 (-10.4 to -6.7)	-6.6 (-9.1 to -4.2)	-8 (-11.5 to -4.5)	-7.7 (-11 to -4.3)	-7.7 (-11.7 to -3.6)	-8.4 (-10.8 to -6)	-4.7 (-6.6 to -2.7)
Observed rate in 2020	0.9	0.76	0.81	0.83	0.98	0.96	1.19	1.09	1.05	1.41	1.02
Expected rate in 2020 (95% CI)	0.87 (0.73 - 1.05)	0.78 (0.66 - 0.92)	1.02 (0.78 - 1.34)	1 (0.84 - 1.19)	0.95 (0.83 - 1.1)	0.96 (0.79 - 1.17)	0.9 (0.68 - 1.19)	0.88 (0.67 - 1.14)	0.91 (0.66 - 1.25)	0.81 (0.67 - 0.98)	0.98 (0.84 - 1.14)
Observed vs expected rate ratio (95% CI)	1.03 (0.86 - 1.2)	0.98 (0.8 - 1.15)	0.79 (0.66 - 0.93)	0.83 (0.69 - 0.97)	1.02 (0.86 - 1.18)	1 (0.84 - 1.16)	1.32 (1.13 - 1.51)	1.24 (1.06 - 1.43)	1.15 (0.98 - 1.32)	1.74 (1.51 - 1.97)	1.04 (0.88 - 1.2)
Rates are shown in suicides per 100,000 person-years. N = 116 individuals who died of suicide with missing data on age were not included in the age-specific analyses.											

© 2021 Sakamoto H et al. *JAMA Network Open.*

eFigure 1. Observed and Expected Monthly Number of Individuals Who Died of Suicide in January Through November, 2011 to 2020, by Occupational Status Category

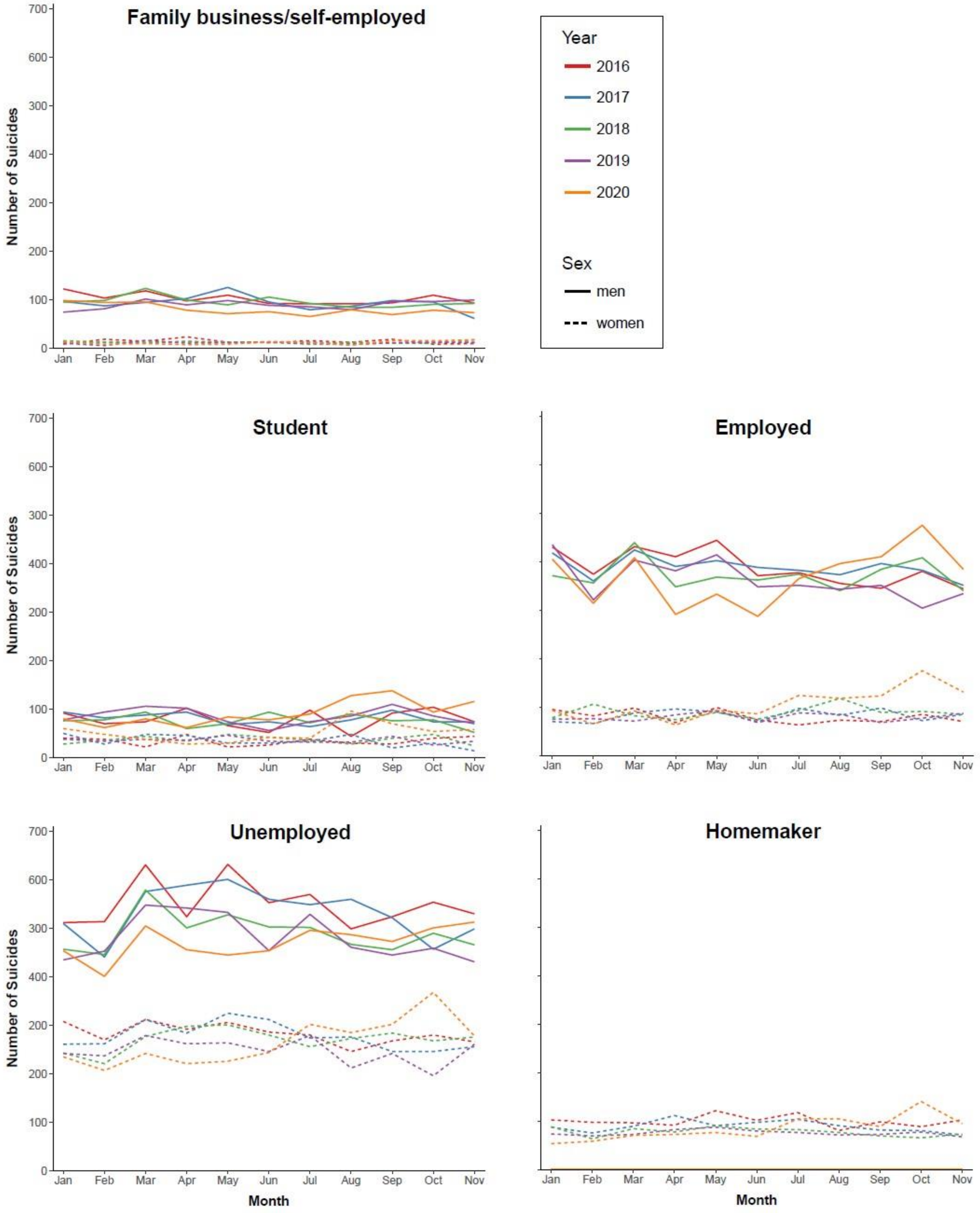

© 2021 Sakamoto H et al. *JAMA Network Open.*

eFigure 2. Observed and Expected Monthly Suicide Rates in January to November, 2011 to 2020, in All Ages
Light colored areas represent 95% CI for expected suicide rates and dots represent the observed rates

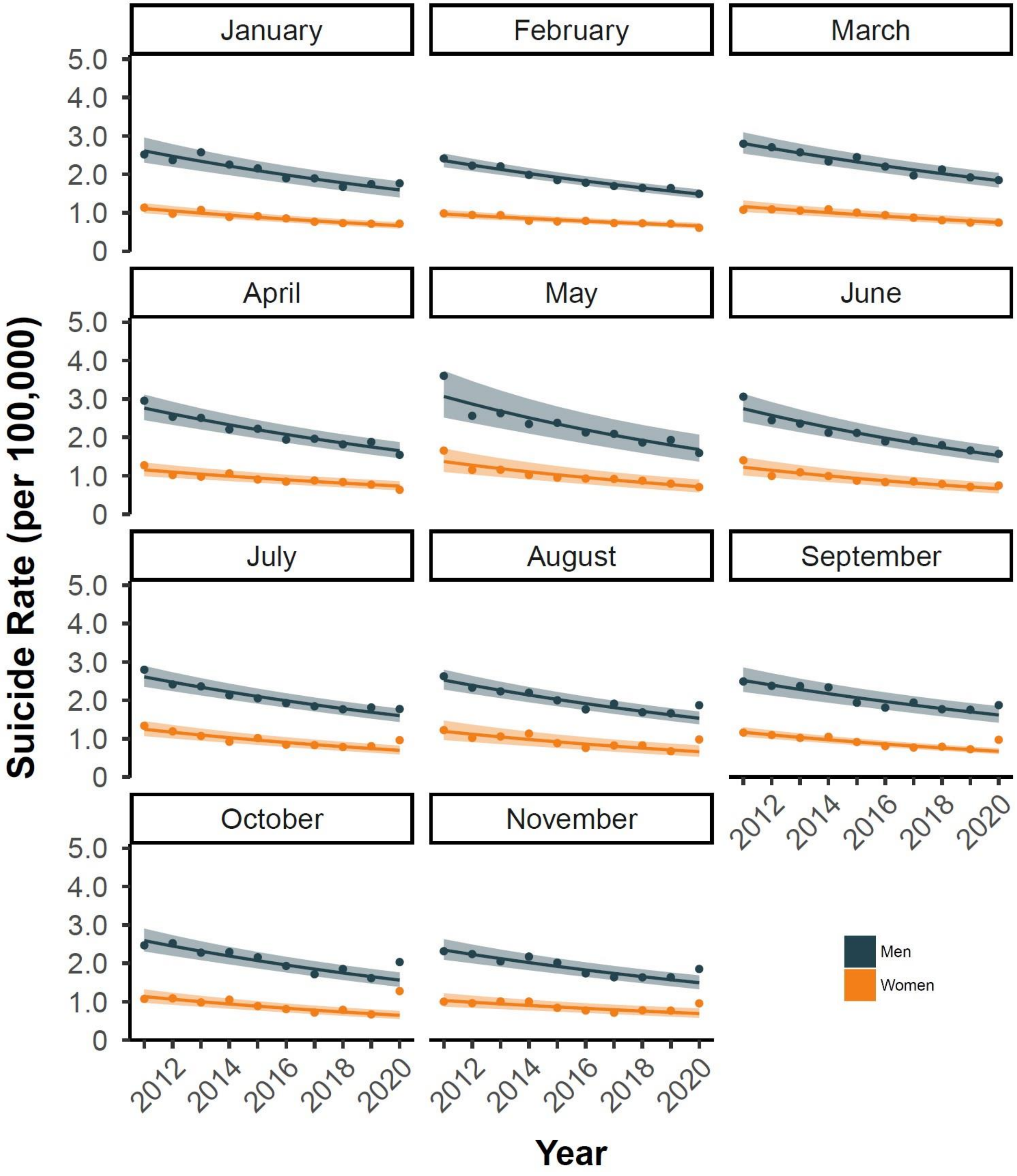

© 2021 Sakamoto H et al. *JAMA Network Open.*

eFigure 3. Observed and Expected Monthly Suicide Rates in January to November, 2011 to 2020, Among Individuals Aged Younger Than 30 Years

Light colored areas represent 95% CI for expected suicide rates and dots represent the observed rates

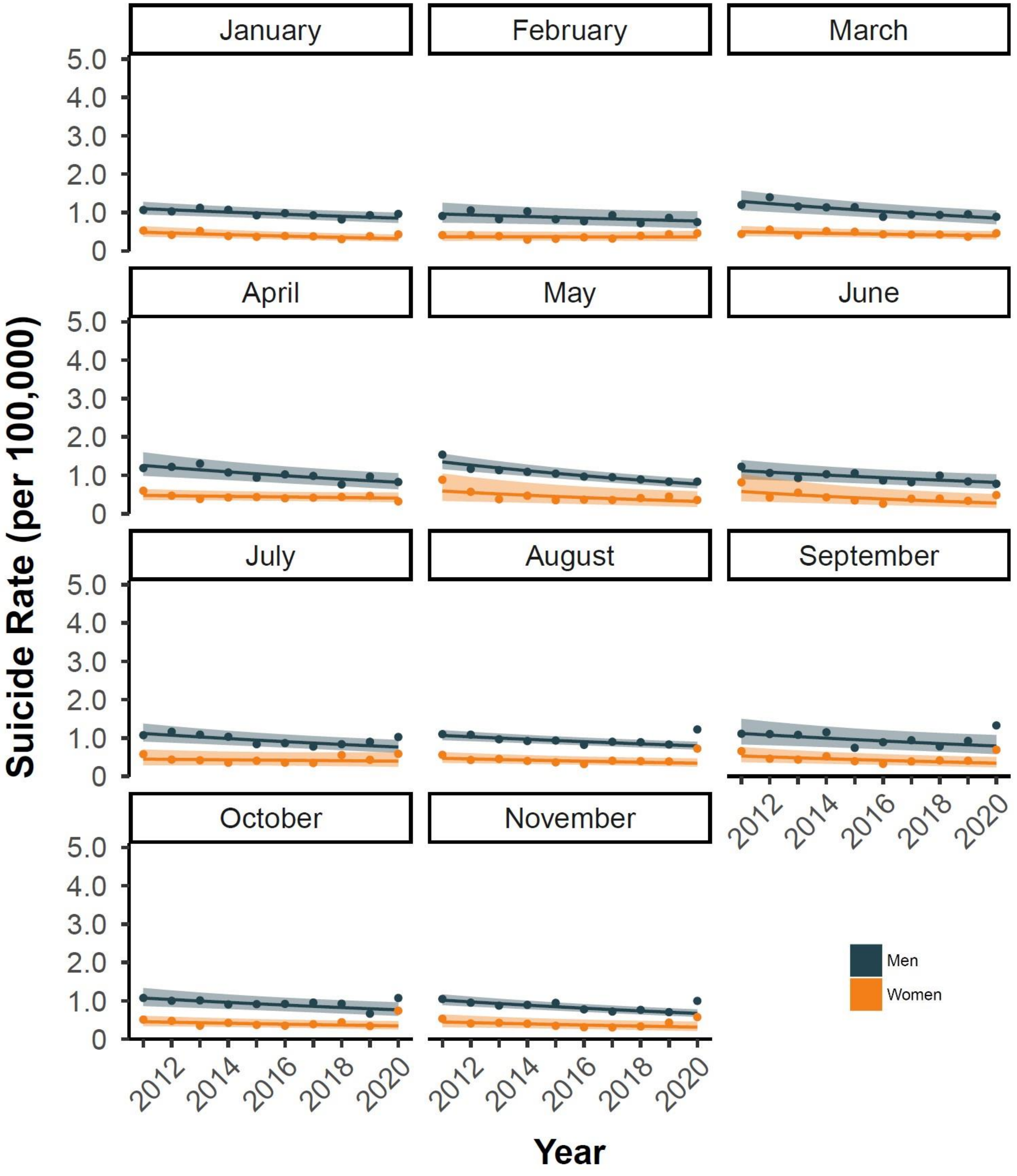

© 2021 Sakamoto H et al. *JAMA Network Open.*

eFigure 4. Observed and Expected Monthly Suicide Rates in January to November, 2011 to 2020, Among Individuals Aged 30 to 49 Years

Light colored areas represent 95% CI for expected suicide rates and dots represent the observed rates

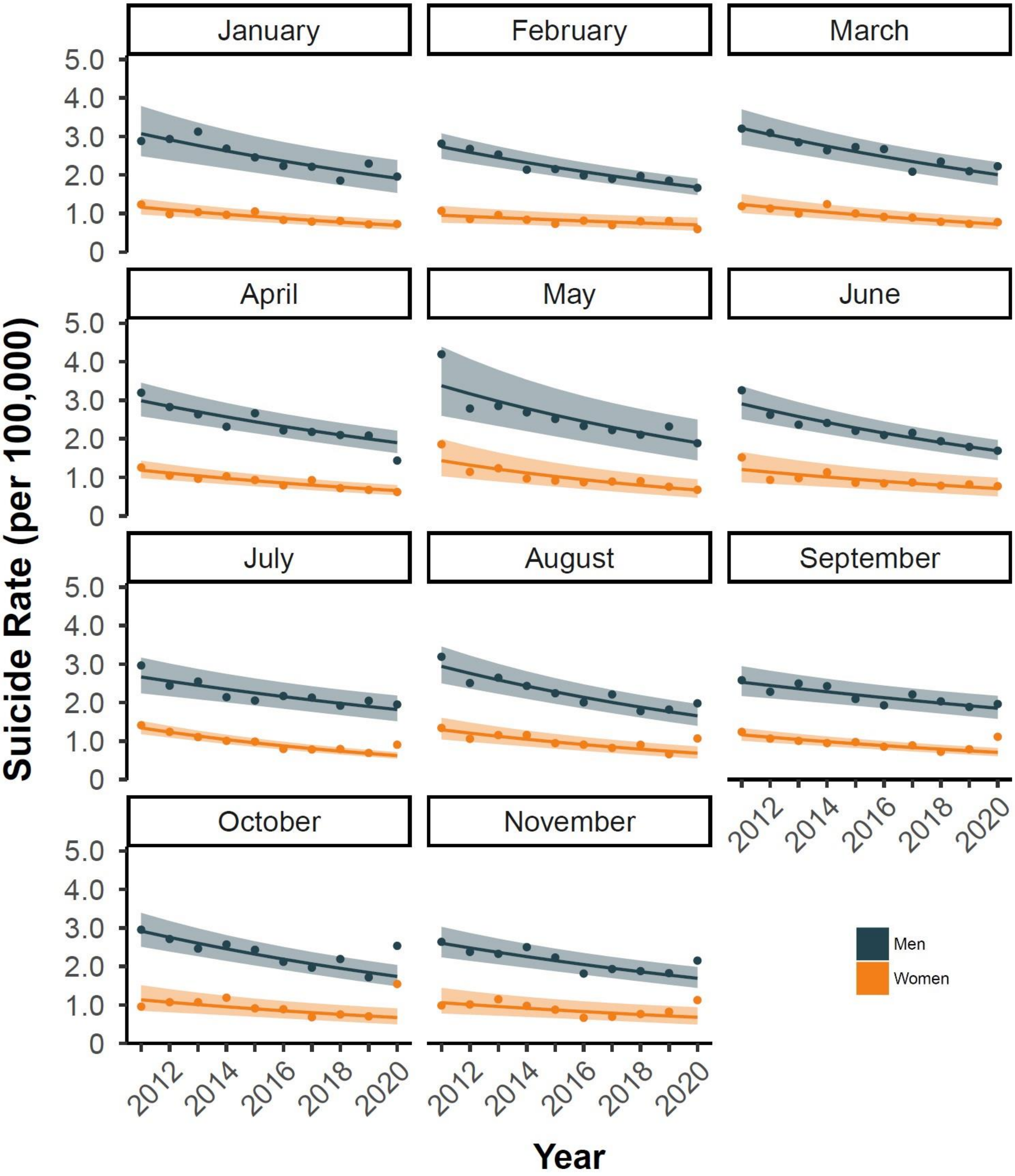

© 2021 Sakamoto H et al. *JAMA Network Open.*

eFigure 5. Observed and Expected Monthly Suicide Rates in January to November, 2011 to 2020, Among Individuals Aged 50 to 69 Years

Light colored areas represent 95% CI for expected suicide rates and dots represent the observed rates

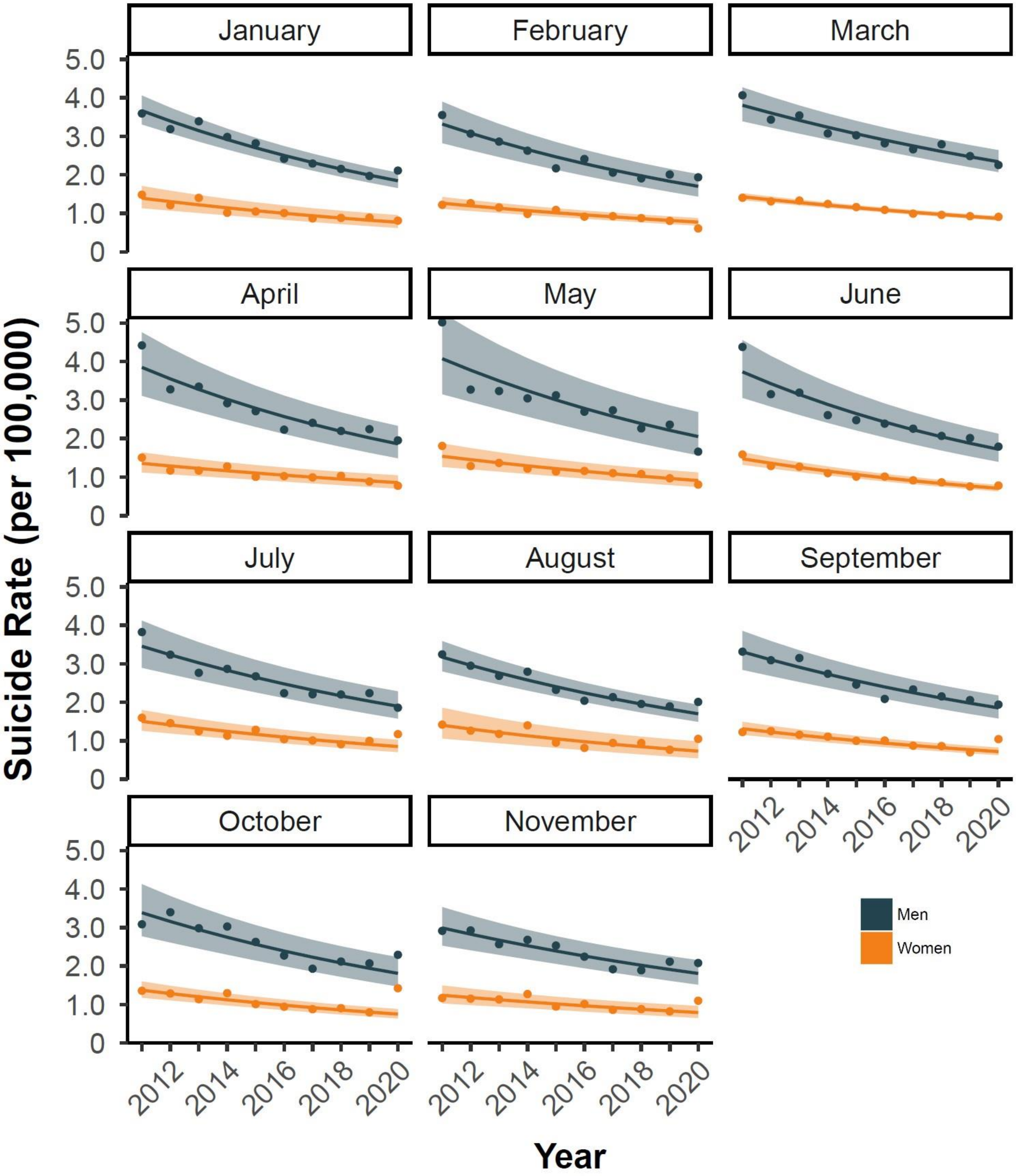

© 2021 Sakamoto H et al. *JAMA Network Open.*

eFigure 6. Observed and Expected Monthly Suicide Rates in January to November, 2011 to 2020, Among Individuals Aged 70 Years or Older
Light colored areas represent 95% CI for predicted suicide rates and dots represent the observed rates

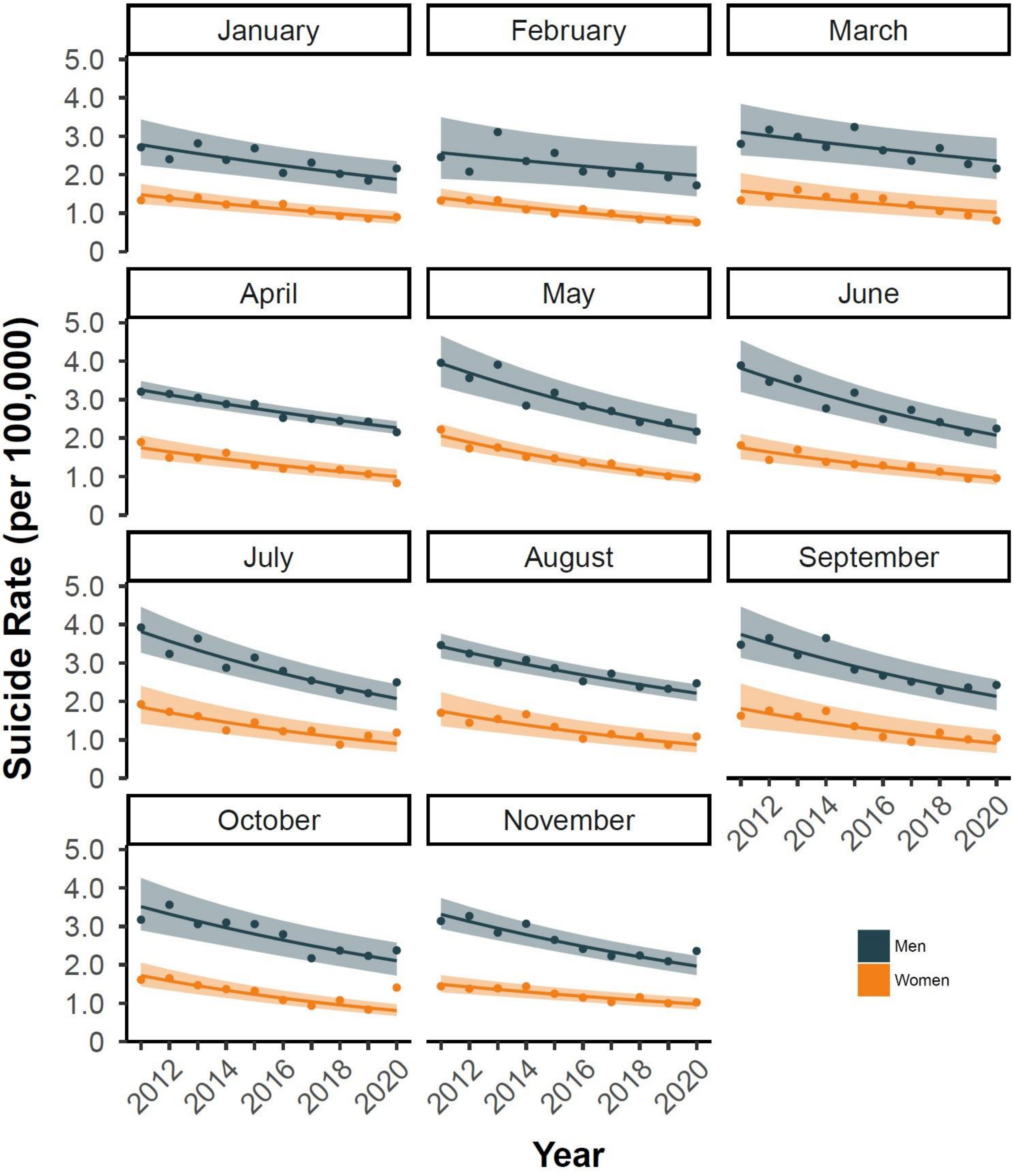

© 2021 Sakamoto H et al. *JAMA Network Open.*
